# Epidemiology and Diagnostic Accuracy of Respiratory Pathogens in Pediatric Populations: Insights From Global Studies

**DOI:** 10.7759/cureus.68652

**Published:** 2024-09-04

**Authors:** Irina Bulata-Pop, Bianca Simionescu, Bogdan Bulata, Lia Monica Junie

**Affiliations:** 1 Microbiology, “Iuliu Hatieganu" University of Medicine and Pharmacy, Cluj-Napoca, ROU; 2 Pediatrics, "Iuliu Hatieganu" University of Medicine and Pharmacy, Cluj-Napoca, ROU; 3 Pediatrics, Emergency Clinical Hospital for Children, Cluj-Napoca, ROU; 4 Microbiology, "Iuliu Hatieganu" University of Medicine and Pharmacy, Cluj-Napoca, ROU

**Keywords:** systematic review, original article, children, lower respiratory infections, etiology

## Abstract

Lower respiratory tract infections (LRTIs) are the most common cause for going to the doctor’s at pediatric age. Respiratory infections are still of interest because they are widespread, significantly impact public health by potentially leading to pandemics, drive antimicrobial resistance through antibiotic misuse, more often spread globally due to traveling, and benefit from ongoing advancements in diagnostics and research for better management. This paper’s main aim was to offer a systematic review of the literature published over the last 10 years on the etiology of LRTIs. The search strategy was based on reviewing original articles, systematic reviews, position papers, and guidelines published in MEDLINE, EMBASE, Cochrane Library, and PubMed. The review was previously registered with PROSPERO. The final review included 27 articles that met the eligibility criteria (studies identifying the etiology of inferior respiratory infections in children, according to the WHO definition, published in the last 10 years). Statistical analysis was performed using Microsoft Excel Version 2406 (Microsoft Corporation, Redmond, Washington, USA) and SPSS Statistics V.23 (IBM Corp., Armonk, New York, USA). The total number of patients was 2,193,978. Eight articles focused on children younger than five years, and two included children under the age of two. The results revealed that *Mycoplasma pneumoniae* and respiratory syncytial virus (RSV) are significant respiratory pathogens with seasonal peaks and age-specific prevalence and that nasopharyngeal aspirates (NPAs) are more reliable than throat swabs for confirming infections due to their higher positive predictive value (PPV). The impact of COVID-19 interventions led to reduced infections from RSV, adenovirus, and influenza viruses, but an increase in rhinovirus post-reopening, with high co-infection rates. Co-infections are common, particularly with pathogens like human bocavirus (HBoV) and RSV, underscoring the need for comprehensive diagnostic approaches. The impact of non-pharmaceutical interventions during the COVID-19 pandemic significantly reduced the prevalence of many respiratory pathogens, except for rhinovirus, which increased post-reopening. Understanding these dynamics is crucial for managing respiratory infections, especially in pediatric populations.

## Introduction and background

Although the causes of respiratory infections in children are generally known in theory, the subject is still of interest for scientists due to the imperfect ways of diagnosing and identifying the etiology, mainly because pneumonia remains the most frequent cause of death in children under five years old [1].

Lower respiratory tract infections (LRTIs) are defined as infections affecting the airways below the larynx or the pulmonary alveoli. They include bronchiolitis, bronchitis, and pneumonia. The symptoms are not specific, including fever, coughing, wheezing, difficulty or rapid breathing, and chest pain. The diagnosis is clinical, based on the physical examination on which rales or condensation are found, alongside changes in the pattern of breathing. In pneumonia, chest X-rays may help in making the difference between possible etiologies [2]. Culture, rapid tests, serology, or polymerase chain reaction (PCR) constitute the methods used for identifying the etiology of the infection [3]. However, PCR, which is the most useful of all tests due to its rapidity of results, is also the most expensive, making it an effective tool for severe cases but not for most LRTIs. Even though the recent COVID-19 pandemic brought closer the use of PCR and rapid tests on a larger scale, they remain costly for hospitals with a large turnover of patients. Overuse of antibiotics generates resistance, a fact proven by numerous infections that do not react to the first line of therapy, especially in developing countries. Preventive methods are extremely important in the battle against the high mortality of LRTIs. Breastfeeding for a minimum of six months has a positive effect on reducing respiratory infections and diarrhea, especially in low-income countries [4]. One of the most cost-effective, the supplementation of vitamin D was proven to be effective [5] and has gained a lot more importance since the COVID-19 pandemic. However, the pandemic impact on childhood immunization was more detrimental than the plusses it brought. According to WHO, 20.5 million children in 2022 and 24.4 million children in 2021 missed out on one or more vaccines. Despite the slight improvement, compared to the number of 18.4 million children who missed out in 2019 before pandemic-related disruptions [6], there is a great need for ongoing catch-up, recovery, and system-strengthening efforts.

The aim of our paper is to identify studies on the etiology of LRTIs and perform a systematic review of the literature published over the last 10 years. Based on this information, our goal was to identify what are the possible causes of LRTIs and design a protocol of diagnosis based on clinical and biological tests that would reduce the anxiety of medical staff and parents, as well as cut down some costs of unnecessary testing, unneeded antibiotic use, and hospitalization.

## Review

Our search strategy was based on reviewing original articles, systematic reviews, position papers, and guidelines published. We identified the review question, and we established the keywords for the search. MEDLINE, EMBASE, Cochrane Library, and PubMed were searched. There was a language restriction applied: only full papers were available in English. After identifying the papers in full text, duplicates were removed, and inclusion criteria were applied.

Inclusion criteria were settled: studies identifying the etiology of inferior respiratory infections in children, published in the last 10 years. We excluded all papers that did not have a full text available, including those that studied both inferior and superior respiratory infections, risk factors for infection, or associations with other pathologies such as asthma, tuberculosis, cancer, etc. For the remaining articles, we checked references for the other articles. 

The population we targeted was formed out of children aged 0 to 18 years of age. LRTI was defined [6] as an infection in the lower respiratory tract (airways below the level of the larynx, including the trachea and alveolar sacs). From a clinical point of view [1], LRTI can take the form of tracheitis, bronchitis, pneumonia, or bronchiolitis and is characterized by the presence of cough and fever, signs of difficulty breathing, and rales or diminished murmur on examination. Pneumonia had to be defined by x-ray alterations. 

The studies were assessed regarding design, methodological quality, levels of evidence, and description. Reviewers independently assessed the risk of bias using the Critical Appraisal Skills Program (CASP). This tool was instrumental in identifying potential biases like selection or publication biases within the included studies. To mitigate these, we used comprehensive search strategies that included multiple databases and gray literature, like unpublished studies, conference abstracts, dissertations, and clinical trial registries. We applied clear and predefined inclusion and exclusion criteria. We acknowledge the language bias. However, the included studies offer a global perspective on the subject. Preferred Reporting Items for Systematic Reviews and Meta-Analyses (PRISMA) checklists were used for the validation of the studies included. The checklist contained ten questions that every article had to answer, regarding whether the aims were clearly stated, whether the qualitative methodology, the design of the research, the recruitment strategy, data collection, and data analysis were adequately formulated if the relationship between researcher and participants was taken into consideration, if there were ethical issues, if the findings were clearly stated, and how valuable the research is. The remaining 27 articles that met the CASP criteria and checked the PRISMA checklist were included. 

This review was registered in the PROSPERO international database of prospectively registered systematic reviews in health in March 2022, with ID CRD42022309946.

The literature screening and data extraction were performed independently by two reviewers, with subsequent cross-checking to ensure accuracy. Any disagreements were resolved through discussion, and if consensus could not be reached, a third reviewer was involved to mediate. The following data were collected: general information on the study-publishing date, geographical region of interest, number of patients included, means of testing for etiology, and findings regarding the etiology of infection.

**Figure 1:**
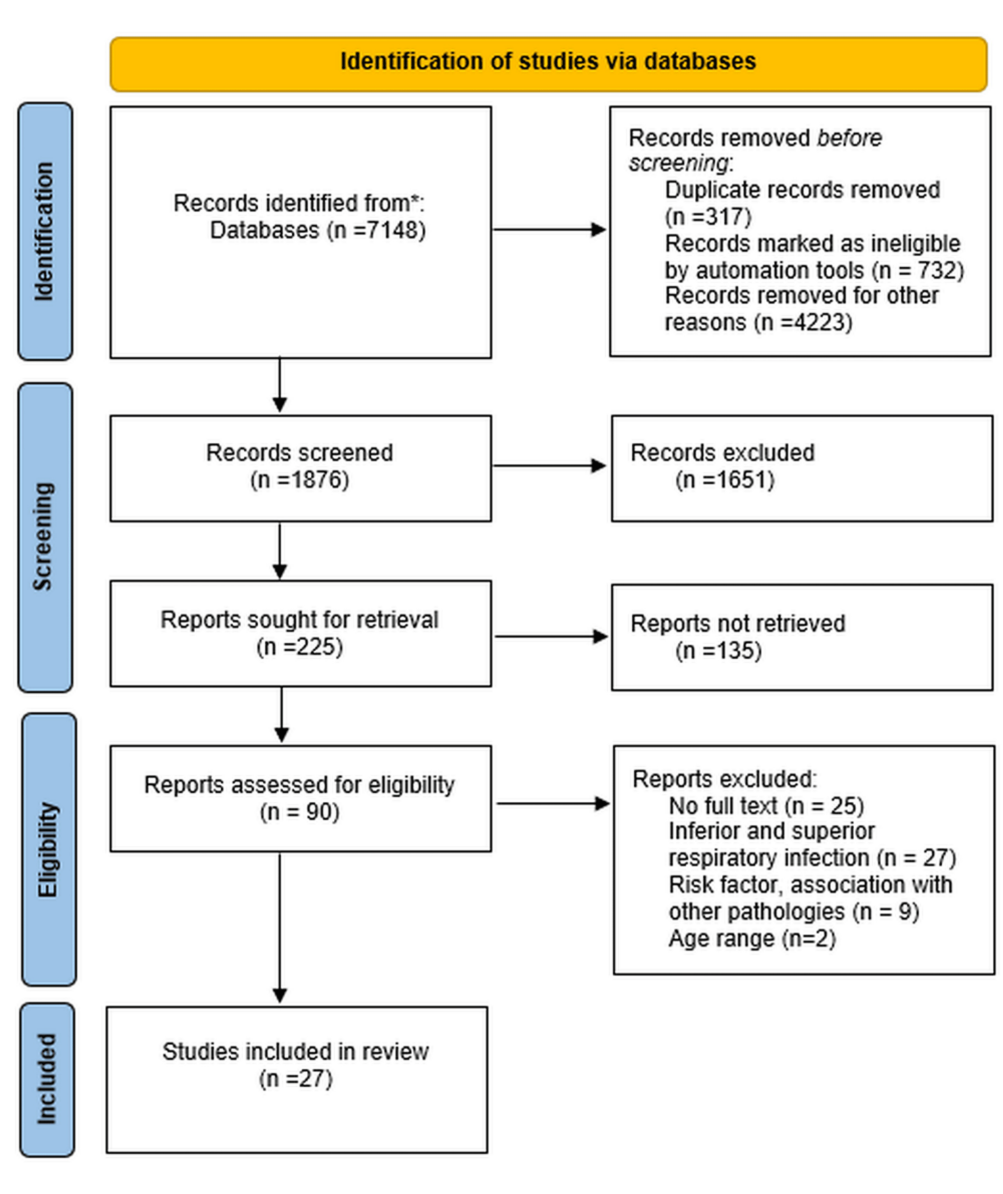
PRISMA flow chart of the selection process. PRISMA: Preferred Reporting Items for Systematic Reviews and Meta-Analyses. Paper selection from MEDLINE, EMBASE, Cochrane Library, PubMed databases, and English literature.

Statistical analysis was performed using Microsoft Excel Version 2406 (Microsoft Corporation, Redmond, Washington, USA) and SPSS Statistics V.23 (IBM Corp., Armonk, New York, USA). Sensitivity, specificity, positive predictive value (PPV), and negative predictive value (NPV) were calculated to compare the efficacy of diagnostic tools. McNemar's test was used to compare paired proportions. Cohen's kappa statistic was applied to assess the agreement between diagnostic tools. Fisher's exact test was conducted to analyze the associations between clinical manifestations and etiological agents in co-infection studies. Figure 1 presents the search strategy for original articles and reviews in the databases mentioned earlier.

Table 1 offers information on the articles [3,7-32], the year of publication, the age range of patients, and the technique and method used for diagnostics. 

**Table 1:** Articles included in the review. NPA: nasopharyngeal aspirate; BAL: bronchoalveolar lavage; PCR: polymerase chain reaction; RT-PCR: reverse transcription polymerase chain reaction; LRTI: lower respiratory tract infection; CAP: community-acquired pneumonia; HRV: human rhinovirus; RSV: respiratory syncytial virus; HMPV: human metapneumovirus; HCoV: human coronavirus; HPIV: human parainfluenza virus, NPS: nasopharyngeal swab; OPS: oropharyngeal swab; LA: lupus anticoagulant.

SI. No	Citation	Authors	Title	Journal	Year of publication	Country	Age range	Specimen
1	[3]	Chou et al.	Comparisons of etiology and diagnostic tools of lower respiratory tract infections in hospitalized young children in Southern Taiwan in two seasons	J Microbiol Immunol Infect	2016	Taiwan	0-5Y	NPS
2	[7]	Sitthikarnkha et al.	Epidemiology of acute lower respiratory tract infection hospitalizations in Thai children: a 5-year national data analysis	Influenza Other Respir Viruses	2022	Taiwan	0-18Y	NPS, OPS, blood
3	[8]	Lei et al.	Viral etiology and epidemiology of pediatric patients hospitalized for acute respiratory tract infections in Macao: a retrospective study from 2014 to 2017	BMC Infect Dis	2021	China	0-13Y	NPS
4	[9]	Chen et al.	Immunoglobulin M profile of viral and atypical pathogens among children with community acquired lower respiratory tract infections in Luzhou, China	BMC Pediatr	2019	China	0-14Y	NPS, blood
5	[10]	Berce et al.	Clinical and laboratory characteristics of viral lower respiratory tract infections in preschool children	Wien Klin Wochenschr	2015	Austria	0-6Y	NPS
6	[11]	Haataja et al.	Hospital admissions for lower respiratory tract infections in children born moderately/late preterm	Pediatr Pulmonol	2018	Finland	0-7Y	NPS, blood
7	[12]	Chen et al.	Acute lower respiratory tract infections by human metapneumovirus in children in Southwest China: a 2-year study	Pediatr Pulmonol	2010	China	0-15.9Y	NPS
8	[13]	Sarna et al.	Viruses causing lower respiratory symptoms in young children: findings from the ORChID birth cohort	Thorax	2018	Australia	0-2Y	NPS
9	[14]	Dierig et al.	*Mycoplasma pneumoniae* detection in children with respiratory tract infections and influence on management: a retrospective cohort study in Switzerland	Acta Pediatr Int J Pediatr	2020	Switzerland	0-10Y	NPS
10	[15]	Kogoj et al.	Prevalence, genotyping and macrolide resistance of *Mycoplasma pneumoniae* among isolates of patients with respiratory tract infections, central Slovenia, 2006 to 2014	Eurosurveillance	2015	Slovenia	0-16Y	NPS, blood
11	[16]	Rodman Berlot et al.	*Mycoplasma pneumoniae* P1 genotype indicates severity of lower respiratory tract infections in children	J Clin Microbiol	2021	Slovenia	0-15Y	NPS
12	[17]	Zar et al.	Aetiology of childhood pneumonia in a well vaccinated South African birth cohort: a nested case-control study of the Drakenstein Child Health Study	Lancet Respir Med	2016	South Africa	0-2Y	NPS
13	[18]	Wadilo et al.	Viral etiologies of lower respiratory tract infections in children < 5 years of age in Addis Ababa, Ethiopia: a prospective case–control study	Virol J	2023	Ethiopia	0-5Y	NPS, OPS
14	[19]	Ueno et al.	Age-specific incidence rates and risk factors for respiratory syncytial virus-associated lower respiratory tract illness in cohort children under 5 years old in the Philippines	Influenza Other Respir Viruses	2019	Philippines	0-5Y	NPS
15	[20]	Vong et al	Acute lower respiratory infections in ≥5-year-old hospitalized patients in Cambodia, a low-income tropical country: Clinical characteristics and pathogenic etiology	BMC Infect Dis	2013	Cambodia	0-5Y	NPS, blood
16	[21]	Lu et al.	Diagnostic value of nasopharyngeal aspirates in children with lower respiratory tract infections	Chin Med J	2017	China	0-17Y	NPS, LA
17	[22]	Bhuyan et al.	Bacterial and viral pathogen spectra of acute respiratory infections in under-5 children in hospital settings in Dhaka city	PLoS One	2017	Bangladesh	0-5Y	NPS
18	[23]	Kang et al.	Etiologic diagnosis of lower respiratory tract bacterial infections using sputum samples and quantitative loop-mediated isothermal amplification	PLoS One	2012		0-18Y	Sputum
19	[24]	Hoffmann et al.	Viral and atypical bacterial etiology of acute respiratory infections in children under 5 years old living in a rural tropical area of Madagascar	PLoS One	2012	Madagascar	0-5Y	NPS
20	[25]	Cebey-López et al.	Viral co-infections in pediatric patients hospitalized with lower tract acute respiratory infections	PLoS One	2015	Spain, England	0-18Y	NPS
21	[26]	Schlaberg et al.	Viral pathogen detection by metagenomics and pan-viral group polymerase chain reaction in children with pneumonia lacking identifiable etiology	J Infect Dis	2017	USA	0-5Y	NPS, OPS
22	[27]	Jain et al.	Community-acquired pneumonia requiring hospitalization among U.S. children	N Engl J Med	2015	USA	0-18Y	NPS
23	[28]	Tsitsiklis et al.	Lower respiratory tract infections in children requiring mechanical ventilation: a multicentre prospective surveillance study incorporating airway metagenomics	The Lancet Microbe	2022	USA	0-17Y	OPS, LA
24	[29]	O'Brien et al.	Causes of severe pneumonia requiring hospital admission in children without HIV infection from Africa and Asia: the PERCH multi-country case-control study	Lancet	2019	Asia and Africa	0-18Y	NPS, OPS, LA, blood culture
25	[30]	Ebruke et al.	The etiology of pneumonia from analysis of lung aspirate and pleural fluid samples: findings from the Pneumonia Etiology Research for Child Health (PERCH) study	Clin Infect Dis	2021	Asia and Africa	0-18Y	NPS, OPS, LA, blood culture
26	[31]	Liu et al.	Impact of COVID-19 pandemic on the prevalence of respiratory viruses in children with lower respiratory tract infections in China	Virol J	2021	China	0-18Y	NPS, LA
27	[32]	Liu et al.	Epidemiology of respiratory pathogens in children with lower respiratory tract infections in Shanghai, China, from 2013 to 2015	Jpn J Infect Dis	2018	China	0-18Y	NPS, LA

From the 27 selected papers, two are epidemiological studies [7,29], two are clinical studies [17,27], two are retrospective cohort studies [12,14], two are diagnostic accuracy studies [21,22], two are molecular and genetic studies [16,23], two focus on public health and vaccination [20,31], and two are multicenter or collaborative studies [8,10].

All selected papers met the definition for LRTIs [33] and provided the protocol for testing. All children admitted to the hospital in the selected period, who met the case definition, were enrolled in the study, achieving the adequacy of patient selection. The rate of detection for each study ranged in the interval accepted in the literature (47% and 95%).

The demographic characteristics of the study populations across the various articles provided insights into the prevalence and impact of respiratory pathogens in children. The studies covered multiple geographical locations, age groups, and seasons, providing a comprehensive overview of respiratory infections in pediatric populations.

The studies predominantly focused on children under the age of five years, with specific age subgroups such as infants (under one year), toddlers (one to three years), and preschool-aged children (three to five years). For example, the study of Lei et al. [8] had a particular age range from 0 to 13 years, while the study of Liu et al. [32] included children under 24 months for RSV prevalence and older children for *Mycoplasma pneumoniae* infections. Dierig et al. [14] found the median age of children with *Mycoplasma pneumoniae* to be 6.4 years, indicating a slightly older pediatric group. The research encompassed a diverse range of geographical settings, including urban areas like Shanghai [32], China, and rural regions such as Ampasimanjeva and Madagascar [24]. Studies were conducted in various countries, including China [8,9,12,21,31,32], Switzerland [14], Slovenia [15], the United States [26-28], Madagascar [24], and other locations. Gender distribution was generally balanced, although some studies reported slight variations. For instance, the study from the Children's Hospital of Fudan University in Shanghai [32] included 300 boys and 188 girls among the 488 children enrolled.

Seasonal trends were significant in the studies [32], particularly for viral infections like RSV, which peaked in winter, and *Mycoplasma pneumoniae*, which peaked in autumn in Shanghai. Many studies [8,11,20,29] focused on children with severe acute respiratory infections requiring hospitalization, such as those conducted at the Children's Hospital of Fudan University [32]. The Madagascar study [24] included both outpatient and hospitalized children, highlighting a broad range of disease severity.

We compared nasopharyngeal aspirates (NPAs) and bronchoalveolar lavage fluids (BALFs) to assess pathogen detection rates in children with lower respiratory tract infections (LRTIs). *Streptococcus pneumoniae *was detected in BALF at a rate of 2.6% in children aged three years or younger and 1.7% in those older than three years. *Mycoplasma pneumoniae* was much more common, with detection rates of 36.6% in the younger group (≤3 years) and 68.7% in the older group (>3 years). This difference between age groups was statistically significant (p < 0.05). Parainfluenza Virus Type 3 was found in 4.6% of the younger children and 0.7% of the older ones, while Adenovirus was detected in 4.1% of children aged three years or younger and in 1.0% of those older than three years. 

**Figure 2:**
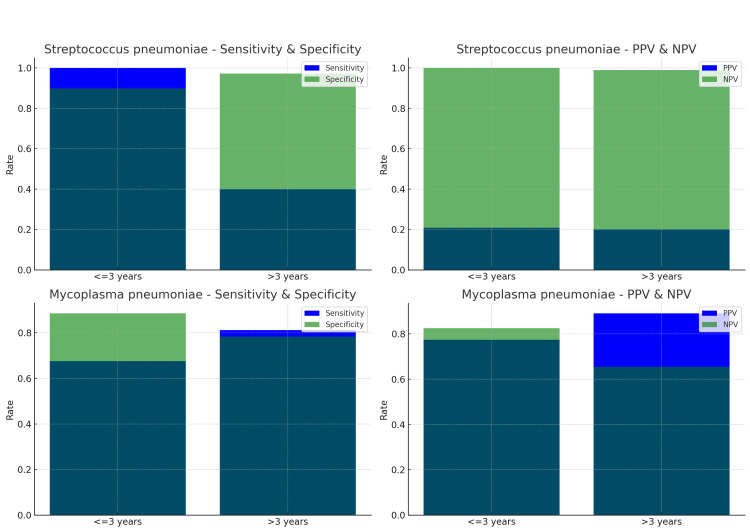
Diagnostic accuracy for NPA vs BALF. Source: References [14-16]. PPV: positive predictive value, NPV: negative predictive value, NPA: nasopharyngeal aspirates, Sp: *Streptococcus pneumoniae*, Mp: *Mycoplasma pneumoniae*. For Sp, NPA is reliable for ruling out infection in children ≤3 years due to high sensitivity and NPV. For Mp, NPA shows moderate to high sensitivity and specificity across all age groups, making it a relatively reliable diagnostic tool, though not without risks of false positives and negatives.

The plots in Figure 2 provide a visual representation of the diagnostic accuracy metrics for nasopharyngeal aspirates (NPAs) compared to bronchoalveolar lavage fluids (BALFs) for *Streptococcus pneumoniae* and *Mycoplasma pneumoniae*.

Table 2 summarizes the positive predictive value (PPV) and negative predictive value (NPV) for nasopharyngeal aspirates (NPA) and throat swabs for detecting *Streptococcus pneumoniae*, *Mycoplasma pneumoniae*, and RSV, calculated using the sensitivity, specificity, and a fixed prevalence rate.

**Table 2:** Positive predictive value (PPV) and negative predictive value (NPV) for nasopharyngeal aspirates (NPAs) and throat swabs. Source: References [14-16]. For all pathogens, the PPV is higher for NPA compared to throat swabs, meaning that a positive result from an NPA is more likely to correctly identify the presence of the pathogen than a positive result from a throat swab.

Pathogen	NPA PPV	NPA NPV	Throat PPV	Throat NPV
Streptococcus pneumoniae	64.0%	97.7%	43.8%	96.4%
Mycoplasma pneumoniae	48.6%	98.2%	35.7%	96.8%
RSV	66.7%	98.8%	40.0%	95.3%

For *Streptococcus pneumoniae* and RSV, NPAs should be preferred due to their higher PPV and slightly better NPV, which makes them more reliable for both ruling in and ruling out these infections. For *Mycoplasma pneumoniae*, both methods are reasonably reliable for ruling out the infection (high NPV), but NPAs offer a higher PPV, making them a better choice for confirming the presence of the pathogen. Out of the total number of patients (2,193,978), only 3% of them had an identified etiology. 

For the under five-year-old patients, the most frequent etiology was viral. RSV was identified as the major cause of bronchiolitis, with adenovirus following alongside influenza virus. It was observed that all these infections have a seasonal presence in the community, occurring mainly in winter and spring or, according to geographical differences, in the first rainy season (March to May). Other viral agents identified were enterovirus, parainfluenza, and human metapneumovirus (hMPV). These factors were mentioned only in a few studies [12,18,21,24-26] because testing for them is not easily available. In what concerns the bacterial etiology [7,8,13,22], *Streptococcus pneumoniae *was the most frequent cause of LRTIs.

**Figure 3:**
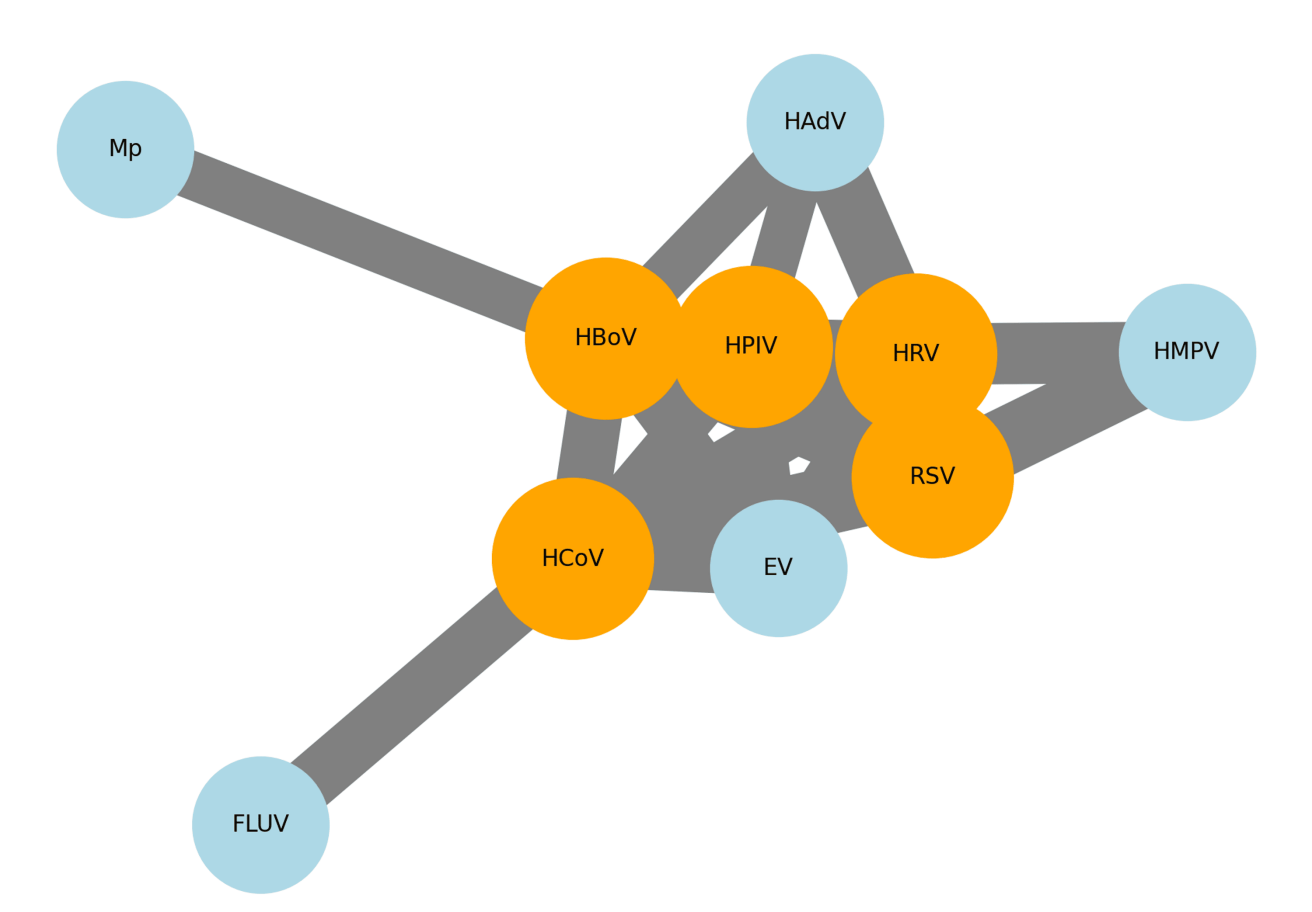
Co-infection network graph. Source: References [22,24,25]. Each node represents a pathogen, with primary agents highlighted in orange and co-infection partners in light blue. The thickness of the edges indicates the strength of the co-infection relationship, based on the number of co-infections observed.

In the studies concerning children above five years of age [7,8,12,14-16,20,21,23,27,29,30], the viral etiology is more equally distributed among RSV, rhinovirus, influenza, hMPV, and adenovirus. The range of bacteria detected in these patients varies between countries-according to the vaccination available: in developing countries, the most frequent cause of pneumonia remains *Streptococcus pneumonia*, tuberculosis, and for the most severe cases, *Haemophilus influenzae* and gram-negative bacteria like *Klebsiella pneumonia* and *Pseudomonas aeruginosa*. In developed countries, *Streptococcus pneumonia* was identified as the cause of pneumonia. The co-infection network graph (Figure 3) visualizes the relationships between primary viral agents and their co-infection partners.

The co-infection analysis [22,24,25] reveals that certain pathogens, particularly human bocavirus (HBoV) and respiratory syncytial virus (RSV), have high rates of co-infection with other respiratory pathogens, as seen in Table 3.

**Table 3:** Co-infection analysis. Source: References [22,24,25]. HRV: human rhinovirus, HBoV: human bocavirus, HCoV: human coronavirus, HPIV: human parainfluenza virus, RSV: respiratory syncytial virus, EV: enterovirus, HMPV: human metapneumovirus, HAdV: human adenovirus, Mp: *Mycoplasma pneumoniae*, FLUV: influenza virus.

Primary viral agent	Co-infection partners	Co-infection counts	Total infections	Co-infection rate
HRV	('EV', 'HBoV', 'HCoV', 'HPIV', 'RSV', 'HMPV', 'HAdV')	32	61	52.4%
HBoV	('HRV', 'HCoV', 'EV', 'HPIV', 'HAdV', 'HMPV', 'RSV', 'Mp')	25	18	138.8%
HCoV	('HPIV', 'HRV', 'HBoV', 'RSV', 'EV', 'FLUV')	29	37	78.3%
HPIV	('HCoV', 'HRV', 'HBoV', 'RSV', 'HAdV', 'EV')	24	35	68.5%
RSV	('HRV', 'HBoV', 'HCoV', 'HPIV', 'HMPV')	35	35	100.0%

For only 17,716 patients (0.8%), the symptoms (cough, fever, and difficulty breathing) were recorded. However, these are somehow implied by the definition of LRTI [33], making it not necessarily to be reported separately. Considering the evolution of the disease, 16.5% of them received medical care in the ICU, while 20% died of the complications.

The studies [15,16,18,24] provided a comprehensive overview of respiratory pathogen epidemiology, highlighting *Mycoplasma pneumoniae* and RSV as significant pathogens with seasonal peaks and age-specific prevalence. The impact of COVID-19 on respiratory infections [31] was evident, with a decrease in RSV, adenovirus, and influenza viruses due to NPIs but an increase in rhinovirus post-reopening. The study of Jain et al. [27] emphasized the need for localized epidemiological data to guide treatment. Diagnostic comparisons showed nasopharyngeal aspirates (NPAs) had higher positive predictive value (PPV) and negative predictive value (NPV) compared to throat swabs, making them more reliable for confirming infections. High co-infection rates, especially with HRV, HBoV, and RSV, were noted, complicating diagnosis and treatment. Studies of Dierig et al. [14] and Rodman Berlot et al. [16] provided insights into *Mycoplasma pneumoniae* detection and genotypic differences in severity, while the research of Hoffmann [24] highlighted the prevalence and seasonal variation of viral pathogens in acute febrile children.

Given the fact that respiratory infections, in general, and LRTIs, in particular, are responsible for most of the hospital admissions in children [33], with the potential of becoming severe illnesses, knowing the etiology is a decisive factor in establishing the best treatment and obtaining healing. In achieving this, it is vital to develop and use a good diagnostic test [3], which would offer high sensitivity in the shortest time possible, for the information to be used immediately in the process of establishing the best treatment, based on evidence.

An important difference observed between developing and developed countries regarding the bacterial etiology of pneumonia concerns pathogens preventable by vaccination. As reported by several authors [17,20,22], in developing countries, infections caused by *Haemophilus influenzae*, *Streptococcus pneumoniae,* and tuberculosis usually take an important toll on children, causing severe illnesses and leaving sequelae. According to the review published in 2000 by McCracken [33], only 25% to 30% of the cases of bacterial pneumonia in developed countries are caused by *Streptococcus pneumonia*. 

Regarding etiology distribution according to age, the group of patients under five years of age [18,19,22,24,32] present more frequently with viral infections caused by RSV, under the form of bronchiolitis. Above five years [8,14,20], children were more often admitted for pneumonia caused by atypical bacteria (Mycoplasma, Chlamydia), with *Streptococcus pneumonia* occurring equally among younger and older patients. Diagnostic tool comparisons showed that NPAs have higher accuracy compared to throat swabs, making them preferable for confirming infections.

Studies from various countries provided valuable insights into pathogen prevalence, seasonal variations, and the challenges of managing respiratory infections in pediatric populations. Geographical variations can influence the results of studies on LRTI in children due to a range of factors. Firstly, different regions may have varying prevalences of specific pathogens causing LRTIs. These differences can be due to local climate conditions, population immunity, and the presence of specific pathogen strains. For instance, regions with higher humidity may see different seasonal patterns in viral infections compared to arid regions. A second factor is the availability and quality of healthcare services, including vaccination programs, access to antibiotics, and healthcare infrastructure, which differ widely between regions. In high-income countries, early diagnosis and treatment might lead to better outcomes, whereas in low- and middle-income countries, delayed treatment due to limited access to healthcare could result in higher morbidity and mortality rates. Socioeconomic status and environmental conditions, also factor in. For example, children in urban areas with high pollution levels may be more susceptible to respiratory infections compared to those in rural areas with cleaner air. Lastly, cultural practices, such as the use of traditional medicines or differing attitudes toward vaccination, can also influence LRTI outcomes. Although this review focuses on literature published in the last 10 years, the time periods during which studies on LRTIs are conducted can also have a substantial impact on their findings due to changes in pathogen epidemiology, as we have experienced during the COVID-19 pandemic, advancements in medical technology, like the optimization of the PCR technique used now, and shifts in public health policies. However, geographical and temporal variations in LRTI study results enhance understanding of disease dynamics, enable targeted interventions and resource allocation, broaden scientific knowledge, and encourage innovation in public health strategies.

Even though nowadays our main concern remains the SARS-CoV-2 infection, old viral infections start to remerge, causing less impressive, but still dangerous infections [31]. For instance, during the influenza season of 2022-2023, 28% of the symptomatic illnesses reported by the CDC [34] belonged to the 5-17 years group, a percentage 5-10% higher than the 2019-2020 season, with almost 700 deaths. The impact of the COVID-19 outbreak [35] has touched various levels in the development of children, regarding physical and behavioral fields.

The COVID-19 pandemic's impact on respiratory infections was a significant focus, with studies [31,35] noting reduced infections due to non-pharmaceutical interventions (NPIs) like social distancing and mask-wearing. This was particularly evident in the study of Liu et al. [31], where significant reductions in RSV, adenovirus, and influenza virus infections were observed, while rhinovirus infections increased post-reopening.

We are worried about the reduction in immunization, vitamin deficiencies [36] due to indoor time, the resurge of respiratory infections [6] that had a declining tendency, like invasive group A Streptococcal disease, measles, and whooping cough, and the severity of the disease [37]. Furthermore, there was a direct impact on the daily routine and relationships among children [38] and their peers and extended family, which might be seen only in the future.

Strengths and limitations

The search strategy was meant to include all the studies on the etiology of LRTIs, but we cannot be sure we did not omit some papers. Although the subject is of great interest, from a scientific and clinical point of view, generally, studies do not limit themselves to strictly targeting the etiology. Putting etiology in the context of risk factors or association with background pathology is common, but these studies did not meet the inclusion criteria for our review. Another limitation concerns the way the study was designed. A lot of the papers concentrate only on viral etiology, which is the most researched area due to the availability of testing methods and the perspective of continuous development of PCR methods.

On the other hand, we included in the review studies done in different parts of the world, with different means, but the conclusions were comparable. If we collate the results on age groups, all the papers included highlight what we already knew about etiology. What is new comes from the difference in occurrence during the year, the relationship with the vaccination policy, and the means of testing; rapid tests are of great use in parts of the world where molecular biology is still out of reach or too expensive. 

Clinical implications

First, this review underlines the importance of localized epidemiological data in guiding effective treatment and prevention strategies for respiratory infections in children. It also underscored the complexity of diagnosing respiratory pathogens due to frequent co-infections, particularly involving HRV, HBoV, and RSV. The research highlighted the importance of vaccination as a means of reducing morbidity and resistance to antibiotics. In countries where there is a strong vaccination policy, the most common LRTIs are caused by viruses, compared to developing countries where bacterial LRTIs are prevalent, forcing doctors to increase the use of antibiotics [37], mainly without proper testing, increasing consequently the antibiotic resistance.

Secondly, this sort of age group profile is useful for outpatients. Protocols based on this information are used to decrease unnecessary invasive therapy strategies.

## Conclusions

Even though the recent pandemic changed a lot in the way we prevent, test, and treat LRTIs, we are still confronted with the same old pathogens for which our methods of testing remain imperfect. The truth revealed again by our study is that identifying an etiology for lower respiratory infections in children is very difficult. An extended study of LRTI etiology and improving the way we obtain the specimens used for testing would be important in giving the right view of the pathology and, most importantly, on the use of antibiotics based only on clinical examination. By accurately describing age groups and their etiology, doctors would make an informed decision based on evidence, rather than on personal experience and fear.
